# Bone Marrow and Adipose Tissue Adenosine Receptors Effect on Osteogenesis and Adipogenesis

**DOI:** 10.3390/ijms21207470

**Published:** 2020-10-10

**Authors:** Anna Eisenstein, Shlok V. Chitalia, Katya Ravid

**Affiliations:** 1 Department of Dermatology, Yale School of Medicine, New Haven, CT 06520, USA; 2 Department of Medicine and Whitaker Cardiovascular Institute, Boston University School of Medicine, Boston, MA 02118, USA; shlokvipulc@gmail.com (S.V.C.); kravid@bu.edu (K.R.)

**Keywords:** adenosine receptors, adipogenesis, osteogenesis, adipose tissue, bone marrow, obesity

## Abstract

Adenosine is an extracellular signaling molecule that is particularly relevant in times of cellular stress, inflammation and metabolic disturbances when the levels of the purine increase. Adenosine acts on two G-protein-coupled stimulatory and on two G-protein-coupled inhibitory receptors, which have varying expression profiles in different tissues and conditions, and have different affinities for the endogenous ligand. Studies point to significant roles of adenosine and its receptors in metabolic disease and bone health, implicating the receptors as potential therapeutic targets. This review will highlight our current understanding of the dichotomous effects of adenosine and its receptors on adipogenesis versus osteogenesis within the bone marrow to maintain bone health, as well as its relationship to obesity. Therapeutic implications will also be reviewed.

## 1. Introduction

**Figure 1:**
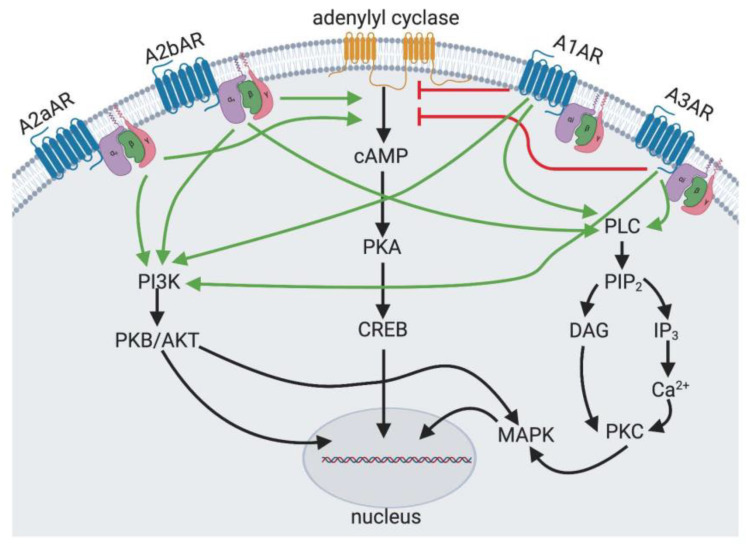
Adenosine Receptor Signaling. The A2aAR and the A2bAR are adenylyl cyclase stimulating receptors through Gαs subunit. The A1AR and the A3AR are adenylyl cyclase inhibitory receptors through Gαi subunit. The A1AR, A3AR and A2bAR also act through phospholipase C (PLC) to increase cytosolic calcium and diacylglycerol (DAG) to activate protein kinase C (PKC) and MAP kinase (MAPK) signalling. All adenosine receptors are capable of activating phosphatidylinositol 3-kinase (PI3K).

Adenosine is a nucleoside that is released by all cells, including adipocytes in vitro and the subcutaneous adipose tissue in vivo [1,2]. Adenosine is formed by the degradation of adenosine triphosphate (ATP), adenosine diphosphate (ADP) and adenosine monophosphate (AMP). Levels of adenosine rise in response to metabolic stress, tissue injury, hypoxia and inflammation [3]. Adenosine acts broadly via four receptors. The A1 adenosine receptor (A1AR) and the A3 adenosine receptor (A3AR) are coupled to Gi, which inhibits adenylyl cyclase and decreases cytosolic levels of cyclic AMP (cAMP). The A2a and the A2b adenosine receptors (A2aAR and A2bAR, respectively) are coupled to the stimulatory G alpha protein (Gs), activate adenylyl cyclase and increase cytosolic levels of cAMP. The adenosine receptors may also act on other signaling cascades (Figure 1). The expression of these receptors and the receptor affinity for adenosine allow for modulation of the effect of adenosine in different physiologic states and in different tissues. As such, the action of adenosine may be contradictory depending on these various factors [3].

## 2. Adenosine Receptors Are Expressed on Bone Marrow-Derived Mesenchymal Stem Cells and Preadipocytes

Adenosine is found extracellularly under pathologic situations including hypoxia, ischemia or cell damage. Cells release adenosine into the extracellular space through equilibrative nucleoside transporters (ENTs). Adenosine can also be formed by the conversion of extracellular ATP, released from damaged cells, by ectonucleoside triphosphate diphosphorylase (CD39) and ecto-5′-nucleotidase (CD73) enzymes. Adenosine can also be metabolized to inosine by adenosine deaminase or phosphorylated to AMP by adenosine kinase. As one can imagine, the coordination of adenosine release is well regulated at baseline and, during times of cellular stress, can become a signal of disequilibrium [4]. Similarly, while adenosine receptors are present on many different cell types and found generally throughout the body, the level of expression varies in different cell types, pathologic states and developmental stages [4,5], and hence the response to adenosine can be, at times, contradictory. The A1AR and A3AR are more ubiquitously expressed in most tissues, while the A2aAR and the A2bAR have more selective expression [6]. The A1AR is expressed at high levels in the brain, spinal cord, adrenal glands and the heart, with slightly lower expression in the liver, bladder, adipose tissue and testis [7,8]. Highest expression of the A3AR occurs in the lung and liver but is also expressed in brain, testis, spleen, thyroid, kidney, bladder, heart, eosinophils and mast cells [7,8]. The A2aAR is most highly expressed in the brain, spleen, thymus, leukocytes and platelets; this receptor is also expressed in moderate levels in the heart, lung and vasculature [7,8]. The A2bAR is expressed in the vasculature of large intestine, ovaries, testis, liver, spleen, adipose tissue, muscle, pancreas, brain, lung, heart, kidneys, eye and lung [6].

The expression of adenosine receptors is increased in various pathologic conditions (review in [9,10]). The A1AR is induced by oxidative stress [11] and hypoxia [12]. The A3AR was found to be upregulated in rheumatoid arthritis and during methotrexate treatment (which increases adenosine levels) as well as in breast and colon cancer [13,14,15,16]. The A2aAR is upregulated in inflammatory states [17,18], hypoxia [19,20] and cellular stress such as during food restriction [21]. The A2aAR was also upregulated in individuals receiving methotrexate therapy [16]. Similarly, the A2bAR is upregulated by inflammation, hypoxia and extracellular adenosine [22,23,24,25]. Expression of the A2bAR is upregulated in conditions of inflammation or hypoxia in the vasculature, intestine, kidneys, heart and lung [9,19,26,27]. Not only does the variable and inducible expression of adenosine receptors indicate the complexity of adenosine receptor signaling, it also indicates the consideration that must be taken when designing therapeutics.

Bone-marrow-derived mesenchymal stem cell (MSCs), precursors to adipocytes and osteoblasts, express the A2bAR and the A2aAR [28]. In testing the activity of adenosine receptors in vitro and in vivo, numerous agonists and antagonists have been developed (Table 1). In Ob1771 preadipocytes, the nonspecific adenosine receptor agonist, 5′-N-Ethylcarboxamidoadenosine (NECA), increased cAMP levels, while the A2aAR specific agonist, CGS 21680 (3-(4-(2-((6-amino-9-((2R,3R,4S,5S)-5-(ethylcarbomoyl)-3,4-dihydroxytetrahydrofuran-2-yl)-9H-purin-2-yl)amino)ethyl)phenyl)propanoic acid), did not increase cAMP levels, which would suggest that the A2bAR is the predominant adenosine receptor in these preadipocytes [29]. All adenosine receptors and adenosine metabolic and catabolic enzymes (adenosine deaminase, adenosine kinase and CD73) are expressed on MSCs (although no A3AR protein was identified by Western blotting), though at different levels and activities [30]. The A2bAR was shown to be the predominant adenosine receptor subtype, based on a cAMP assay [30].

The A1AR is the most highly expressed adenosine receptor in white adipose tissue, followed by the A3AR, A2aAR and A2bAR [31,32,33]. Adipose tissue can be divided into the mature adipocytes and the stromal vascular fraction, which contains preadipocytes as well as endothelial cells and fibroblasts. The stromal vascular fraction from mice and humans expresses the A2bAR [34]. In vitro, adipocyte precursors (preadipocytes) have a higher expression of the A2aAR and A2bAR, but, following differentiation to a mature adipocyte, there is greater expression of A1AR [29,34,35,36]. Adenosine receptors are also expressed in adipose tissue. The A2bAR is notably also expressed in other metabolic tissues such as the pancreas, liver and muscle [6,37,38]. Moreover, mice fed a high-fat diet (HFD) have increased expression of the A2bAR in the liver, gastrocnemius muscle and the epididymal visceral adipose tissue [37]. Interestingly, the A2bAR mRNA expression is significantly elevated in the subcutaneous adipose tissue of obese individuals as compared to lean individuals [37]. Of interest, adenosine is released from adipocytes. The adenosine content of abdominal subcutaneous adipose tissue has been measured in obese and lean individuals. The levels show a trend towards increased adenosine levels in the adipose tissue of obese (0.67 pmol/g wet weight) versus lean (0.42 pmol/g wet weight) individuals [39]. Within the bone, osteoclasts express all four adenosine receptors, and mature osteoblasts express A1 and A2bAR [40].

## 3. Regulation of Adipogenesis and Osteogenesis by Bone Marrow Cell Adenosine Receptors

Adipogenesis results from the differentiation of adipocyte precursors into mature adipocytes capable of storing lipid in lipid droplets and secreting adipokines as well as cytokines either within the bone marrow or within adipose tissue depots. Adipocyte precursors are found in the bone marrow and within the stromal vascular fraction of peripheral adipose tissue depots. In the bone marrow, adipocytes differentiate from the MSCs, which are common progenitors that are also capable of differentiating into osteoblasts and chondrocytes. The process of adipogenesis requires a cascade of transcription factors. The master regulator of adipogenesis is the nuclear hormone receptor, peroxisome proliferator-activated receptor gamma (PPARγ). PPARγ is necessary for adipogenesis; mice lacking PPARγ and humans with dominant negative mutations in PPARγ have severe lipodystrophy and are insulin-resistant [41,42]. Moreover, PPARγ expression is sufficient for adipogenesis, as the retroviral expression of PPARγ in cultured fibroblasts resulted in adipogenesis [43]. It is generally thought that in the early stage of adipogenesis, CCAAT enhancer binding protein-beta and -delta (C/EBP-β and C/EBP-δ) induce the expression of PPARγ and C/EBP-α [44,45,46,47].

One of the first studies investigating the role of adenosine receptors in adipogenesis found that the treatment of rat primary preadipocytes with the adenosine analogue, NECA, promoted differentiation [36]. In bone-marrow-derived MSCs, activation of the A2aAR promoted the expression of the above-described adipogenic transcription factors and enhanced adipocyte differentiation, while activation of the A1AR promoted lipogenesis and lipid accumulation in the mature adipocyte [30]. Transfection of the A1AR into a murine osteoblast precursor cell line promoted adipogenesis. In the same model, forced expression of the A2bAR inhibited adipogenesis and promoted osteogenesis [48]. Activation of the A2bAR with BAY 60-6583, an A2bAR-specific agonist, inhibits adipogenesis in ex vivo stromal vascular cells isolated from subcutaneous inguinal adipose tissue [34]. This effect was found to be dependent on the expression of the transcription and differentiation regulator, Krüppel-like factor 4 (KLF4), which was also induced by A2bAR agonism [34]. Notably, while BAY 60-6583 is the most selective A2bAR agonist available, in certain cell lines BAY 60-6583 was reported to act as a partial agonist or even as an antagonist of the A2bAR [49].

Within the bone marrow, there is thought to be a dichotomous relationship between adipogenesis and osteogenesis, whereby the MSC is directed to one lineage or the other. The control of this fate decision is still being investigated. Some research points to adenosine receptors playing a role in dictating the formation of adipocytes or osteoblasts. Clinically, these decisions may manifest during aging, when the bone marrow contains more fat than younger marrow, or in osteoporosis, where there is loss of bone and an accumulation of fat. Transcription factors important for bone development include runt-related gene family members, such as Runx2. Osteoblast differentiation is known to be stimulated by fibroblast growth factor, insulin-like growth factor, bone morphogenic proteins and the Wnt/β-catenin pathway.

There has been interest in the role of adenosine and adenosine receptors in bone metabolism. Using pharmacologic inhibitors and A2aAR knock out (KO) mice, the A2aAR was shown to promote proliferation of bone-marrow-derived MSCs [50]. During osteoblast differentiation, the A2bAR is upregulated in the first few days of differentiation, and then is downregulated [30]. Osteoblast differentiation was greater with the nonspecific adenosine receptor agonist, NECA, and this effect was inhibited by the A2bAR antagonist, MRS1706, but not by the A2aAR antagonist, SCH442416. Additionally, the A2aAR agonist, CGS21680, had no effect on bone differentiation [30]. This all suggests that the A2bAR is the predominant adenosine receptor in augmenting osteoblast differentiation. These findings were supported and advanced in the study by Carroll et al. Using an A2bAR-specific agonist and A2bAR KO mice, the authors showed that activation of the A2bAR promotes osteoblast differentiation [28]. Furthermore, microcomputed tomography analysis of adult femurs from A2bAR KO and wild type (WT) mice showed lower bone density in the KO mice compared to the WT mice [28]. In addition, the A2bAR KO mice had delayed fracture repair as compared to the WT mice [28]. In a study of A2bAR KO from youth to adult age, alterations in bone homeostasis were observed in the KO mice. Most notably, there was a decrease in bone mineral density and trabecular volume as well as reduced formation of new bone in A2bAR KO mice as compared to WT mice [51]. In contrast, the application of an A2bAR agonist reduced bone loss in ovariectomized mice [52], underscoring the need to carefully evaluate the results based on the usage of ligands versus genetic ablation. Regarding the A1AR, pharmacologic inhibition of or genetic knockout of this receptor in mice impairs osteoclast differentiation [53,54]. Furthermore, A1AR KO mice that were ovariectomized, as a model of post-menopausal bone loss, maintained bone volume and had decreased osteoclast bone resorption compared to wild type [55]. Importantly, treatment with an A1AR antagonist prevented ovariectomy-induced bone loss [55]. Conversely, A2aAR agonists have been shown to inhibit osteoclast differentiation and function, and femurs from A2aAR KO mice had decreased bone volume to trabecular bone volume ratio and increased osteoclasts [56]. Thus, the A2bAR and the A2aAR both regulate bone turnover by affecting the osteoblast and osteoclast lineages, respectively. In a study utilizing human bone marrow cells, similar results were found: notably, that inhibition of the A1AR and activation of the A2aAR and A2bAR inhibits osteoclast formation, while A2bAR and A2aAR promote osteoblast formation [57]. 

The ectonucleotidase CD73, which regulates levels of extracellular adenosine through the metabolism AMP to adenosine, plays an important role in bone metabolism. CD73 KO mice have impaired osteoblast differentiation and decreased bone formation with the development of osteopenia [58]. In vitro, this was found to be mediated by the A2bAR, and this is more fully reviewed in the above section [58]. CD73 was also found to be important for bone repair following injury in aged mice [59]. The expression of CD39, which hydrolyzes ATP and ADP, and CD73 as well as adenosine levels in the bone marrow, were found to be decreased in a mouse model of osteoporosis. This same group also found that an A2bAR agonist reduced bone loss in ovariectomized mice [52]. A more recent study reported CD39 on gingiva-derived mesenchymal stem cells as a promoter of osteogenesis through the Wnt/β-catenin signaling pathway [60].

While the above studies focus on the cellular mechanism of osteogenesis, further direct investigation is needed on the effect of adenosine and its receptors on bone density in humans. Due to the beneficial effect of activation of adenosine receptors on bone density in various mice models [61,62,63], Zarebska et al. investigated the influence of exercise endurance on plasma ATP levels with the hypothesis that ATP concentration, a predecessor molecule of adenosine, is likely to correlate with exercise level [63]. This was a one-year study that compared 18 to 34-year old athletes, including futsal players, sprinters and endurance athletes, to a control group. A treadmill test was taken until the participants reached exhaustion and ATP levels were measured. There were higher ATP levels in athletes who had better exercise endurance, postulating a resulting increase in adenosine and adenosine receptor activation. Further studies are warranted to elucidate the contribution of adenosine receptor activation under different physiological conditions, including exercise and with varying bone density.

## 4. Adenosine Receptors in Glucose Homeostasis and Obesity

The function of the A1AR in inhibiting lipolysis has been extensively studied. It is thought that endogenously released adenosine maintains a constant inhibitory signal to adipocyte lipolysis. When adenosine deaminase, which removes endogenous adenosine by degrading it to inosine, is added to the culture of adipocytes from fasted rats, lipolysis reaches maximal levels [64,65]. It has also been shown that adipocytes isolated from humans have a similar response to A1AR agonism, such that treatment with PIA, an A1AR agonist, inhibits isoprenaline-stimulated lipolysis [39]. Conversely, when an A1AR antagonist, 8-Cyclopentyl-1,3-dipropylxanthine (DPCPX), was applied to rat adipocytes, lipolysis increased [66]. A different A1AR antagonist, 8-phenyltheophylline, also increased lipolysis in adipocytes from obese Zucker (fa/fa) rats [67]. When an A1AR agonist was given to Wistar and Zucker fatty rats, levels of free fatty acids, glycerol and triglycerides were reduced [33,68]. These findings were also verified in A1AR KO mice. In these conditions, when an adenosine analog, 2-chloroadenosine, was added to A1AR KO adipocytes, there was no inhibition of lipolysis, as occurred in control WT mice [69]. Furthermore, systemic administration of N-Cyclopentyladenosine (CPA), an A1AR agonist, reduced plasma levels of free fatty acids, glycerol and triglycerides (reducing lipolysis) in WT but not A1AR KO mice. Importantly, A1AR KO mice at baseline had elevated free fatty acids as compared to WT mice, suggesting a tonic-suppressive effect on lipolysis by adenosine through the A1AR in vivo [69]. Furthermore, the body weight of A1AR KO mice was significantly increased by 7–8.5% in older (>5 months) male as compared to controls [69]. Conversely, in aged A1AR KO mice (14–16 months old), the KO mice weighed slightly less than WT age-matched controls, with more lean mass and less abdominal fat mass [70]. Correspondingly, aged A1AR KO mice had better glucose tolerance and insulin sensitivity systemically and within visceral adipose tissue [70]. In an HFD model of obesity, A1AR KO mice were heavier than WT mice, had elevated fasting plasma and insulin levels and had impaired glucose clearance, as determined in hyperinsulinemic-euglycemic clamp studies [71]. Overexpression of the A1AR in the adipose tissue of mice resulted in decreased plasma free fatty acid levels as compared to controls [72]. There was no effect on body weight on a normal chow diet or with HFD challenge. Interestingly, overexpression of the A1AR was protective against insulin resistance [72]. Adipocytes from lean individuals as compared to obese individuals were more responsive to the effect of A1AR agonism on reducing lipolysis. The mechanism for this effect was shown to be a result of a greater ability of A1AR agonist to decrease adenylate cyclase activity in adipocytes from lean individuals. The role of the A1AR on leptin secretion by adipocytes has also been studied [73]. Sprague-Dawley rats were treated with the A1AR-selective agonist, CPA (N6-Cyclopentyladenosine), and serum leptin levels were measured. Interestingly, stimulation of the A1AR increased leptin concentration in the serum, a finding that was not seen in the vehicle-treated rats. The authors contend that this finding was a result of increased secretion of pre-formed leptin, as the mRNA levels of leptin did not change with A1AR activation. This finding would fit with the idea that leptin secretion is negatively regulated by cAMP. A1AR agonism of adipocytes from lean and obese individuals revealed enhanced glucose uptake with A1AR agonism by PIA in adipocytes from lean individuals as compared to obese individuals [39]. During glucose challenge, freely fed A1AR KO mice have increased insulin and glucagon secretion, though no difference was seen in plasma glucose as compared to WT mice [32]. Clearly, A1ARs play an important role in lipid homeostasis, a consideration that should be made when developing therapeutic targets of the A1AR.

The A2bAR has also been studied in the context of lipid and glucose homeostasis. Koupenova et al. showed that lack of the A2bAR in ApoE-deficient mice fed a HFD resulted in elevated liver and plasma cholesterol and triglycerides, hepatic steatosis and atherosclerotic plaques [38]. Furthermore, administration of the A2bAR agonist, BAY 60-6583 (2-[[6-Amino-3,5-dicyano-4-[4-(cyclopropylmethoxy)penyl]-2-pyridinyl]thio]-acetamide) to ApoE-deficient mice on HFD reduced atherosclerotic plaque formation and circulating plasma triglycerides and cholesterol [38]. In a type I diabetes model, the adenosine receptor agonist, NECA, reduced plasma glucose levels and ameliorated the diabetes-induced decrease in pancreatic insulin [74]. In an attempt to determine which adenosine receptor was responsible for this effect of NECA, the authors found that the A1AR agonist, 2-Chloro-N6-cyclopentyladenosine (CCPA), decreased hyperglycemia, but to a lesser extent than that of NECA. The A2aAR agonist, CGS21680, did not alter glucose levels and NECA was able to suppress hyperglycemia in A2aAR KO, suggesting that this receptor was not playing a significant role. When NECA and an A2bAR antagonist, MRS 1754, were injected together, the suppressive effect of NECA was abolished, suggesting that the NECA action through the A2bAR played a dominant role in reducing plasma glucose in this model [74]. In one study, KKAy mice (named after Kyoji Kondo who developed a diabetic mouse strain) were used to model type 2 diabetes mellitus. In this case, administration of the A2bAR antagonist, ATL-801, for two days reduced hepatic glucose production and increased glucose uptake in skeletal muscle and brown adipose tissue [75]. Acute challenge with the adenosine receptor agonist, NECA, delayed glucose disposal and increased fasting glucose levels [75]. This effect was eliminated in A2bAR KO mice, which suggests that NECA activation of the A2bAR impaired glucose uptake and utilization [75]. On the other hand, A2bAR KO mice fed an HFD for 16 weeks have increased adiposity, delayed glucose clearance and impaired insulin sensitivity [37]. Intraperitoneal injection of the A2bAR-specific agonist, BAY 60-6583, during HFD feeding lowered fasting glucose levels and improved glucose and insulin tolerance [37]. Further work showed that restoration of macrophage expression of A2bAR in an otherwise full-body A2bAR KO mouse restored insulin signaling and glucose homeostasis to the levels of WT mice [76]. Furthermore, glucose homeostasis and systemic and tissue inflammatory markers were ameliorated with restoration of A2bAR signaling in macrophages [76]. In a third model, A2bAR KO mice fed a chow diet, but not an HFD, gained more weight and had impaired glucose and lipid homeostasis as compared to WT mice [77]. This effect was shown to be a result of the ability of A2bAR to promote alternative macrophage activation and prevent adipose tissue inflammation [77]. The role of the A2bAR in insulin and glucose sensitivity has been extensively studied in mouse models of obesity and diabetes and appears to play an active role in maintaining glucose homeostasis [37,78].

The A2aAR has also been implicated in adipose tissue dynamics, obesity and inflammation. Mice fed a HFD for 12 weeks had increased expression of the A2aAR in macrophages within the adipose tissue as compared to mice on a low-fat diet [79]. These macrophages were also more proinflammatory (M1) as compared to WT mice [79]. A2aAR KO mice fed an HFD gained more weight than WT mice, despite similar food consumption and energy expenditure [79]. A2aAR KO mice had greater abdominal fat mass, increased adipocyte size than WT mice, greater insulin resistance and glucose intolerance [79,80]. Furthermore, in another study, A2aAR KO mice fed an HFD for 16 weeks had reduced glucose tolerance due to a decrease in insulin secretion by the pancreas rather than a difference in insulin sensitivity [81]. In an HFD model of obesity in Swiss mice, the administration of CGS21680, an A2aAR agonist, improved glucose homeostasis, as determined by insulin tolerance test, and reduced inflammatory markers such as tumor necrosis factor alpha (TNFα) systemically and in the visceral adipose tissue [82]. Overall, activation of the A2aAR may also be a potential therapeutic target in metabolic disease.

An exciting study explored the effect of the A2aAR on brown adipose tissue. The authors found that the A2aAR is the most highly expressed adenosine receptor in brown adipose tissue [31]. A2aAR KO mice or pharmacologic inhibition of the A2aAR decreased thermogenesis [31]. Conversely, the administration of A2aAR agonists increased energy expenditure [31]. This finding was explained by browning of the white adipose tissue which was induced by activation of the A2aAR [31]. Finally, in mice fed an HFD, administration of an A2aAR agonist decreased weight gain and improved glucose tolerance [31]. Ruan et al. showed that A2aAR expression in brown adipose tissue was important in cardiac remodeling during hypertension [83]. A2aAR KO mice had worsened hypertensive cardiac remodeling compared to WT mice in a mouse model of hypertension [83]. The A2aAR was highly induced in the brown adipose tissue in this model, and lack of the A2aAR in KO mice impaired brown adipose tissue thermogenesis induced by the hypertensive model in WT mice [83]. Furthermore, A2aAR-induced thermogenesis in brown adipose tissue was important for secretion of fibroblast growth factor 21 (FGF21), which was necessary to protect against hypertension-induced cardiac remodeling [83]. In a study in lean men using Positron Emission Tomography—Computed Tomography (PET/CT), adenosine was found to increase perfusion in brown adipose tissue to a greater extent than cold exposure [84]. Interestingly, using the PET radioligand, [^11^C]TMSX, which binds specifically to the A2aAR, lean men were found to have reduced brown adipose tissue radioligand binding (a marker of A2aAR receptor density) following cold exposure [84]. However, in mice lacking all four ARs, termed quadruple knockout mice (QKO), body temperature did not differ from WT mice [85], suggesting that ARs are not necessary for baseline body temperature or that the four adenosine receptors may have reciprocal and opposing effects on body temperature such that deletion of one adenosine receptor is balanced by the deletion of a different adenosine receptor. Regardless, the role of the A2aAR in brown adipose tissue appears to be clinically relevant and may lead to future therapeutic efforts.

Gnad et al. evaluated the effect of A2bAR stimulation or suppression in major energy dissipating tissues [86]. Their studies demonstrated a significant expression of A2bAR in skeletal muscle and brown adipose tissues. Upon genetic deletion of the A2bAR specifically in the skeletal muscle of mice, there was loss of muscle mass, exemplified by sarcopenia and reduced energy expenditure. A2bAR activation had opposite effects. Similarly, the deletion of A2bAR specifically in adipose tissue increased age-related reduction in brown adipose tissue, while stimulation of the A2bAR protected brown adipose tissue, and reduced white adipose tissue and obesity. This study demonstrates the beneficial effect of A2bAR stimulation on several cell types important in thermogenesis and in preventing obesity and age-related sarcopenia. Future studies are needed to understand how this receptor stimulation mediates this effect, especially in complex disease models consisting of other pathological processes accompanying obesity, such as diabetes.

## 5. Discussion: Implications for Therapeutic Benefit

Adenosine receptors play a role in regulating adipogenesis in peripheral adipose tissue and the bone marrow, and influence whole-body metabolism in terms of lipid storage and glucose homeostasis. Given the potential therapeutic benefits of adenosine receptors, this is an exciting time in adenosine biology. Given the complex biology and diverse, and, at times, contradictory effects of the adenosine receptors, continued development and study of drugs that target specific adenosine receptors will be key to the ultimate goal of creating therapeutic agents that lack potential adverse influences (thoroughly reviewed in [87]).

As shown in Table 2, several A1AR agonists, including SDZWAG994, ARA and RPR749, have been evaluated in humans as antilipolytic agents for hypertriglyceridemia [88,89,90]. RPR749 is an orally active A1AR agonist. In a double-blind, single increasing dose, placebo-controlled, parallel group randomized study, six parallel groups of eight men (six individuals in the active arm and two in the placebo arm in each group) were given RPR749 or control as a single dose. The average free fatty acid concentration in the serum decreased in all treatment groups [90]. Unfortunately, A1AR agonists have significant side effects, including in the heart and kidney, and also become desensitized after repeated exposure. Partial agonists like GS-9667 (previously known at CVT-3619), decreased plasma free fatty acids without desensitization or significant side effects in rodents [91,92]. In a single ascending dose study, healthy non-obese and obese individuals were given a single oral dose of GS-9667 and, in a multiple ascending dose study, healthy obese subjects received GS-9667 for 14 days. The studies found that GS-9667 resulted in decreased free fatty acid levels in a dose-dependent manner, which was maintained over the 14 days of treatment [93]. In addition to the above-cited side effects identified in these trials, the additional consequences of A1AR agonism in the long term may include effects on bone homeostasis with potential for greater risk of osteoporosis.

Other exciting potential targets include the A2bAR, given its reported role in improving glucose and insulin homeostasis, and the A2aAR, given its reported ability to promote thermogenesis and browning of the white adipose tissue. However, given the potential for alterations in bone metabolism and other off-target effects, caution should be applied when evaluating adenosine receptor modulators. Much effort has been devoted to the synthesis of therapeutic drugs that target adenosine receptors, but several clinical trials have failed, and few such drugs are approved. Adenosine itself and regadenoson, an A2aAR agonist, are both approved for coronary stress imaging and for the treatment of paroxysmal supraventricular tachycardia in the case of adenosine. The first adenosine receptor antagonist to be FDA-approved is the A2aAR antagonist istradefylline (Nourianz), which is used with levodopa/carbidopa in Parkinson’s disease [94]. While istradefylline had a good safety profile in over 4000 patients tested with this drug, a different A2aAR antagonist led to death from agranulocytosis in five patients in a phase III clinical trial [95]. An ongoing review of the approved A2aAR antagonist will be necessary to determine if there are long-term consequences, including impairment in bone or adipose tissue metabolism.

## Figures and Tables

**Table 1:** Experimental agonists and antagonists of the adenosine receptors (see the abreviation section for drugs full names).

Receptor	Agonist (+)/Antagonist (−)	Drug Acronym
A1AR	+	CPA, PIA, CCPA
A1AR	−	DPCPX
A2aAR	+	CGS 21680
A2aAR	−	SCH442416
A2bAR	+	BAY 60-6583
A2bAR	−	MRS1706, MRS-1754, ATL-801
A3AR	+	IB-MECA, C1-IB-MECA, MRS5698
A3AR	−	MRS1523, MRS1220
All ARs	+	NECA

**Table 2:** A1AR Modulators Trialed in Humans.

Receptor	Activity	Drug Acronym	Therapeutic Target (Reference)
A1AR	Agonist	SDZWAG994	Hypertriglyceridemia [88]
A1AR	Agonist	ARA	Hypertriglyceridemia [89]
A1AR	Agonist	RPR749	Hypertriglyceridemia [90]
A1AR	Partial agonist	GS-9667	Hyperlipidemia [91]

## Abbreviations

ATP	Adenosine triphosphate
ADP	Adenosine diphosphate
AMP	Adenosine monophosphate
A1AR	A1 adenosine receptor
A2aAR	A2a adenosine receptor
A2bAR	A2b adenosine receptor
A3AR	A3 adenosine receptor
cAMP	Cyclic adenosine monophosphate
DAG	Diacyglycerol
PKC	Protein kinase C
PLC	Phospholipase C
PI3K	Phosphoinositide 3-kinase
MAPK	Mitogen-activated protein kinase
MSC	Mesenchymal stem cell
PPARγ	Peroxisome proliferator-activated receptor gamma
CEBP	CCAAT enhancer binding protein
KLF4	Krüppel-like factor 4
Runx2	Runt-related transcription factor 2
TNFα	Tumor necrosis factor alpha
CPA	N6-Cyclopentyladenosine
PIA	Phenylisopropyladenosine
CCPA	2-Chloro-N6-Cyclopentyladenosine
DPCPX	8-Cyclopentyl-1,3-dipropylxanthine
CGS 21680	3-(4-(2-((6-amino-9-((2R,3R,4S,5S)-5-(ethylcarbomoyl)-3,4-dihydroxytetrahydrofuran-2-yl)-9H-purin-2-yl)amino)ethyl)phenyl)propanoic acid
SCH442416	2-(2-Furanyl)-7-[3-(4-methoxyphenyl)propyl]-7H-pyrazolo[4,3-e][1,2,4]triazolo[1,5-c]pyrimidin-5-amine
BAY 60-6583	2-[[6-Amino-3,5-dicyano-4-[4-(cyclopropylmethoxy)penyl]-2-pyridinyl]thio]-acetamide
MRS-1706	N-(4-Acetylphenyl)-2-[4-(2,3,6,7-tetrahydro-2,6-dioxo-1,3-dipropyl-1H-purin-8-yl)phenoxy]acetamide
MRS-1754	N-4(4-Cyanophenyl)-2-[4-(2,3,6,7-tetrahydro-2,6-dioxo-1,3-dipropyl-1H-purin-8-yl)phenoxy]acetamide
ATL-801	(N-[5-(1-cyclopropyl-2,6-dioxo-3-propul-2,3,6,7-tetrahydro-1H-purin-8-yl)-pyridin-2-yl]-N-ethyl-nicotinamide
IB-MECA	N^6^-(3-Iodobenzyl)adenosine-5′-N-methyluronamide
C1-IB-MECA	2-Chloro-N^6^-(3-Iodobenzyl)adenosine-5′-N-methyluronamide
MRS5698	(1S,2R,3S,4R,5S)-4-[6-[[(3-Chlorophenyl)methyl]amino]-2-[2-(3,4-difluorophenyl)ethynyl]-9H-purin-9-yl]-2,3-dihydroxy-N-methylbicyclo[3.1.0]hexane-1-carboxamide
MRS1523	3-Propyl-6-ethyl-5-[(ethylthio)carbonyl]-2 phenyl-4-propyl-3-pyridine carboxylate
MRS1220	N-[9-Chloro-2-(2-furanyl)[1,2,4]-triazol[1,5-c]quinazolin-5-yl]benzene acetamide
NECA	5′-N-Ethylcarboxamidoadenosine
SDZWAG994	N-Cyclohexyl-2′-O-methyladenosine
ARA	([1S,2R,3R,5R]-3-methoxymethyl-5-[6-(1-[5-trifluoromethyl-pyridin-2-yl]pyrrolidine-3-[S]-ylamino)-purin-9-yl]cyclopentane-1,2-diol)
RPR749	C_22_H_26_F_3_N_7_O_3_
GS-9667	(2S,3S,4R,5R)-2-(((2-fluorophenyl)thio)methyl)-5-(6-(((1R,2R)-2-hydroxycyclopentyl)amino)-9H-purin-9-yl)tetrahydrofuran-3,4-diol
